# Differential neurophysiological correlates of information processing in Internet gaming disorder and alcohol use disorder measured by event-related potentials

**DOI:** 10.1038/s41598-017-09679-z

**Published:** 2017-08-22

**Authors:** Minkyung Park, Yeon Jin Kim, Dai Jin Kim, Jung-Seok Choi

**Affiliations:** 1 Department of Psychiatry, SMG-SNU Boramae Medical Center, Seoul, Republic of Korea; 2 Department of Psychiatry, Seoul St. Mary’s Hospital, The Catholic University of Korea College of Medicine, Seoul, Republic of Korea; 3 Department of Psychiatry and Behavioral Science, Seoul National University College of Medicine, Seoul, Republic of Korea

## Abstract

Internet gaming disorder (IGD) shares clinical and neuropsychological features with alcohol use disorder (AUD), but few studies have identified the neurophysiological characteristics of IGD. We investigated the N100 and P300 event-related potentials (ERPs) in patients with IGD to compare them with those of patients with AUD and healthy controls (HCs). Twenty-six patients with IGD, 22 patients with AUD, and 29 HCs participated in this study. ERPs were acquired from young male adults during an auditory oddball task. Between-group differences in N100 and P300 were investigated separately using repeated-measures analysis of variance. Correlations between the ERP values and neurocognitive functioning of each group were examined. Both the IGD and AUD groups showed reduced P300 amplitudes at the midline central and parietal area compared with the HCs. The IGD exhibited reduced N100 amplitudes at the midline frontal area compared with the HCs. The reduced P300 were correlated with a higher spatial span error rate in the IGD. The reduced N100 and P300 were not correlated with Internet addiction severity scores in the IGD. These results indicate that IGD have abnormalities in the P300 comparable to those in AUD. Moreover, the reduction in N100 could be considered a candidate trait marker for IGD.

## Introduction

Internet gaming disorder (IGD) is defined as excessive or uncontrolled Internet gaming activity that may lead to severe impairment in psychological and social functioning^1^. As the negative consequences of Internet gaming activity have emerged as a significant public health concern, the Diagnostic and Statistical Manual of Mental Disorders, Fifth Edition (DSM-5) has included IGD as a condition for further study, and research about IGD based on psychological, electrophysiological, and neuroimaging techniques has proliferated^2^. IGD has also been conceptualized as a behavioral addiction, characterized by the loss of control over the engagement in the behavior and impulse and the presence of a craving state, similar to gambling disorder or pathological gambling^3–6^. Although IGD does not involve toxic agents, a number of studies have identified that IGD shares clinical features and comorbidities of substance use disorder (SUD) such as tolerance, withdrawal, cravings, and relapse^4, 7, 8^. In addition, previous studies have reported that patients with IGD and alcohol use disorder (AUD), which is one of the most common SUDs, are similar in terms of emotional, temperamental, and personality traits^8^. These characteristics include craving, compulsive engagement, diminished ability to control behavior, failures to regulate impulses, tolerance, withdrawal, and adverse psychosocial consequences^9^. However, despite these similarities, little evidence shows whether IGD and SUD share common neurophysiological features. Therefore, it is worthwhile to examine the electrical activity in the brains of patients with IGD and SUD.

Neuroimaging studies have detected changes in the brain structure of patients with IGD^10, 11^. Some brain regions related to decision making, such as the inferior frontal gyrus (IFG) and anterior cingulate cortex (ACC), have reduced gray matter volume in patients with IGD compared with in healthy controls (HCs)^12–14^. In addition, reduced thickness of the orbitofrontal cortex (OFC), which is implicated in the neurobiological mechanism of drug and behavioral addiction characterized by craving, has been detected in male adolescents with Internet addiction^15^. Kim *et al*.^10^ reported increased regional homogeneity (ReHO) in the posterior cingulate cortex (PCC) of resting-state patients with IGD and AUD. However, ReHo decreased in the superior temporal gyrus (STG) in patients with IGD and in the ACC of patients with AUD^10^.

Electroencephalography (EEG) is a useful tool for assessing brain functioning. EEG is generally believed to reflect the summed postsynaptic potentials in the dendrites of parallel-aligned pyramidal cells oriented perpendicular to the cortical surface. Several studies have used quantitative electroencephalography (QEEG) to assess patients with IGD and reported similarities or differences in the resting-state QEEG of patients with IGD and AUD^16, 17^.

Scalp-recorded event-related potentials (ERPs), derived from the average time-locked EEG in response to sensory, motor, or cognitive events, furnish information about the neuronal activity involved in specific cognitive tasks as a function of time^18^. The P300 wave is a late cognitive ERP component associated with context updating and allocation of attentional resources^19^. The P300 ERP component occurs when subjects are actively involved in detecting rare target stimuli occurring among more frequent standard stimuli during an oddball task^20^. A number of studies have reported that the P300 amplitude decreases in patients with IGD or AUD^21, 22^. One study reported that patients with IGD have lower amplitude of auditory P300 waves than do HCs, and the alteration of P300 amplitudes may be associated with changes in neuronal activity by the excessive exposure to loud auditory stimuli during Internet gaming^22^. In addition, abstinent patients with AUD are widely known to exhibit lower P300 amplitudes compared with HCs in response to deviant stimuli^23^. However, this finding of dysfunctional brain activity in patients with AUD emerged from data from patients >30-years-old who were suffering from chronic alcoholism. On the other hand, the N100 wave is an early sensory ERP component that occurs ~100 ms after stimulus onset. It is thought to reflect mechanisms involved in the initial sensory or attentional selection process and is generated in the primary auditory cortex of the temporal lobe^24–26^. Reductions in the earlier visual N100 component are found at midline central or parietal sites in patients with AUD^27, 28^, which may reflect dysfunctional visual attentional processes in patients with AUD^29^. In contrast, other studies have failed to observe differences in auditory N100 values between patients with AUD and HCs^29, 30^. The inconsistent findings reported by auditory and visual ERP studies may be attributed to the effects of different levels of sensitivity to the task modalities^31^.

In the present study, we directly compared the time-specific ERP components in patients with IGD with those of patients with AUD and HCs employing the ERP technique, particularly in the auditory domain, to elucidate the neurophysiological features of patients with IGD as an addictive disorder. Prior research had demonstrated direct relations between neurocognitive functioning and ERP values, and numerous studies had widely reported amplitudes of P300 reflect impaired attention allocation and working memory capacity^32–35^. However, to date, relations between ERP values and neurocognitive performance in IGD are unexplored. Therefore, we explored relationships between ERP components and neurocognitive functioning in patients with IGD or AUD. As mentioned above, patients with IGD showed characteristics similar to those observed in individuals with AUD^5, 9^, and reduced P300 amplitudes in AUD or IGD have been reported in previous several separate studies^21, 22^. Therefore, we hypothesized that, as a behavioral addiction, IGD might share neurophysiological abnormalities with AUD and predicted that patients with IGD and those with AUD would have lower N100 and P300 amplitudes compared with HCs and that ERP values would be related to neurocognitive functioning in each group.

## Results

### Demographic and clinical data

No group differences in education were observed. However, age (*F*_2, 74_ = 9.129, *p* < 0.001) and estimated IQ scores (*F*_2, 74_ = 6.010, *p* = 0.004) were different among the three groups. The *post hoc* test corrected using the Bonferroni adjustment for multiple comparisons revealed that patients with AUD had lower estimated IQ scores than did HCs (*p* = 0.003), but no differences were observed between other groups (AUD *vs*. IGD, *p* = 0.108, IGD *vs*. HCs, *p* = 0.566). All participants were ≥18 years of age, but patients with AUD were significantly older than those with IGD and HCs (AUD *vs*. IGD, *p* < 0.001, AUD *vs*. HCs, *p* = 0.018, IGD *vs*. HCs, *p* = 0.361). The demographic and clinical characteristics of the participants are presented in Table 1.

**Table 1:** Demographic and clinical characteristics of subjects.

	HCs (n = 29)	IGD (n = 26)	AUD (n = 22)	*F*	*P*	Post hoc
***Demographic data***						
Age (years)	24.66 ± 3.80	22.69 ± 4.76	28.36 ± 5.40	9.129	<0.001***	*H*, *I* < *A*
Education (years)	14.18 ± 1.66	13.28 ± 1.37	14.00 ± 2.08	2.008	0.142	
Estimated IQ	118.93 ± 8.45	114.92 ± 12.36	108.00 ± 12.80	6.010	0.004**	*H* > *A*
***Clinical data***						
IAT	28.50 ± 8.26	76.25 ± 8.54	30.50 ± 7.47	217.864	<0.001***	*H, A* < *I*
AUDIT-K	5.54 ± 4.07	6.19 ± 4.75	24.65 ± 5.23	75.050	<0.001***	*H, I* < *A*
Duration of illness (years)	—	5.94 ± 2.04	4.90 ± 2.41	3.674	0.063	
***Neurocognitive data***						
TMT A RT (sec)	19.14 ± 6.51	22.12 ± 8.26	25.68 ± 22.47	0.481	0.620	
TMT B RT (sec)	53.72 ± 29.19	52.15 ± 22.46	75.23 ± 70.39	0.099	0.906	
Stroop word RT (sec)	53.97 ± 12.15	58.62 ± 14.23	61.00 ± 17.04	1.042	0.358	
Stroop word errors	0.52 ± 0.74	0.50 ± 0.91	0.91 ± 1.60	0.573	0.566	
Stroop color RT (sec)	89.24 ± 14.88	98.15 ± 18.31	111.68 ± 46.61	0.568	0.569	
Stroop color errors	2.34 ± 2.06	1.81 ± 1.63	2.77 ± 4.38	0.419	0.660	
IED total errors	14.24 ± 10.12	14.31 ± 9.42	17.00 ± 11.02	0.178	0.837	
SSP span length	7.97 ± 1.72	8.00 ± 1.36	7.18 ± 1.50	0.357	0.701	
SSP total error	10.00 ± 7.84	8.12 ± 7.49	12.14 ± 5.19	0.690	0.505	
SST proportion of successful stops last sub-block	0.65 ± 0.29	0.52 ± 0.24	0.59 ± 0.24	1.774	0.177	
SST SSRT last sub-block (ms)	139.98 ± 59.41	169.68 ± 78.53	184.02 ± 144.93	0.440	0.646	

AUDIT-K = Korean version of the Alcohol Use Disorder Identification Test; IQ = Intelligence quotient; IAT = Young’s Internet Addiction Test; TMT = Trail Making Test; RT = Reaction Time; IED = Intra-Extra Dimensional Set Shift; SSP = Spatial Span Test; SST = Stop Signal Test; SSRT = Stop Signal Reaction Time.

Data are mean ± standard deviation.

**P < 0.01; ***P < 0.001.

### Behavioral performance

In the auditory oddball task, the button-press reaction time and accuracy rate did not differ among the three groups. Although the AUD group showed a slower response compared with the HCs (395.11 ± 78.67 *vs*. 349.91 ± 41.58 ms), the difference between patients with AUD and HCs was not significant. The behavioral performance of all groups was almost perfect. The AUD group had a lower accuracy rate than did the HCs (95.56 ± 8.94 *vs*. 98.31 ± 3.12%), but the difference was not statistically significant. The behavioral performance data are presented in Table 2.

**Table 2:** Behavioral performance and event-related potential (ERP) values (N100 and P300 amplitudes and latencies) in patients with Internet gaming disorder (IGD) or alcohol use disorder (AUD) and healthy controls (HCs).

	HCs (*n* = 29)	IGD (*n* = 26)	AUD (n = 22)	*F*	*P*	η^2^	Post hoc
***Behavioral results***							
Accuracy rate (%)	98.31 ± 3.12	98.06 ± 3.89	95.56 ± 8.94	0.053	0.948	0.002	
Reaction time (ms)	349.91 ± 41.58	367.06 ± 51.71	395.11 ± 78.67	1.603	0.209	0.044	
***P300 ERP values***							
Cz amplitude (*μ*V)	4.84 ± 2.20	4.17 ± 1.86	3.56 ± 1.43	1.138	0.326	0.031	
CPz amplitude (*μ*V)	6.66 ± 1.65	5.35 ± 1.47	4.95 ± 1.72	5.669	0.005	0.138	*H* > *I, A*
Pz amplitude (*μ*V)	6.49 ± 2.34	5.17 ± 2.06	4.84 ± 1.86	3.925	0.024	0.100	*H* > *I, A*
Cz latency (ms)	337.43 ± 40.08	359.69 ± 53.70	342.64 ± 46.56	1.699	0.190	0.046	
CPz latency (ms)	354.07 ± 40.38	377.84 ± 44.93	366.55 ± 44.46	3.028	0.055	0.079	
Pz latency (ms)	346.62 ± 40.81	365.68 ± 56.88	369.73 ± 55.64	1.451	0.241	0.039	
***N100 ERP values***							
Fz amplitude (*μ*V)	−3.03 ± 1.28	−2.11 ± 1.63	−2.29 ± 1.18	3.056	0.053	0.078	
FCz amplitude (*μ*V)	−2.65 ± 1.24	−1.76 ± 1.32	−1.98 ± 0.92	3.629	0.032	0.095	*H* > *I*
Fz latency (ms)	142.90 ± 25.83	137.03 ± 22.82	130.91 ± 22.57	0.820	0.445	0.022	
FCz latency (ms)	141.10 ± 24.02	136.00 ± 21.69	133.90 ± 21.60	0.687	0.506	0.020	

Data are mean ± standard deviation.

^*^ *P* < 0.05; n.s. = not significant.

### ERP amplitudes and latencies

**Figure 1:**
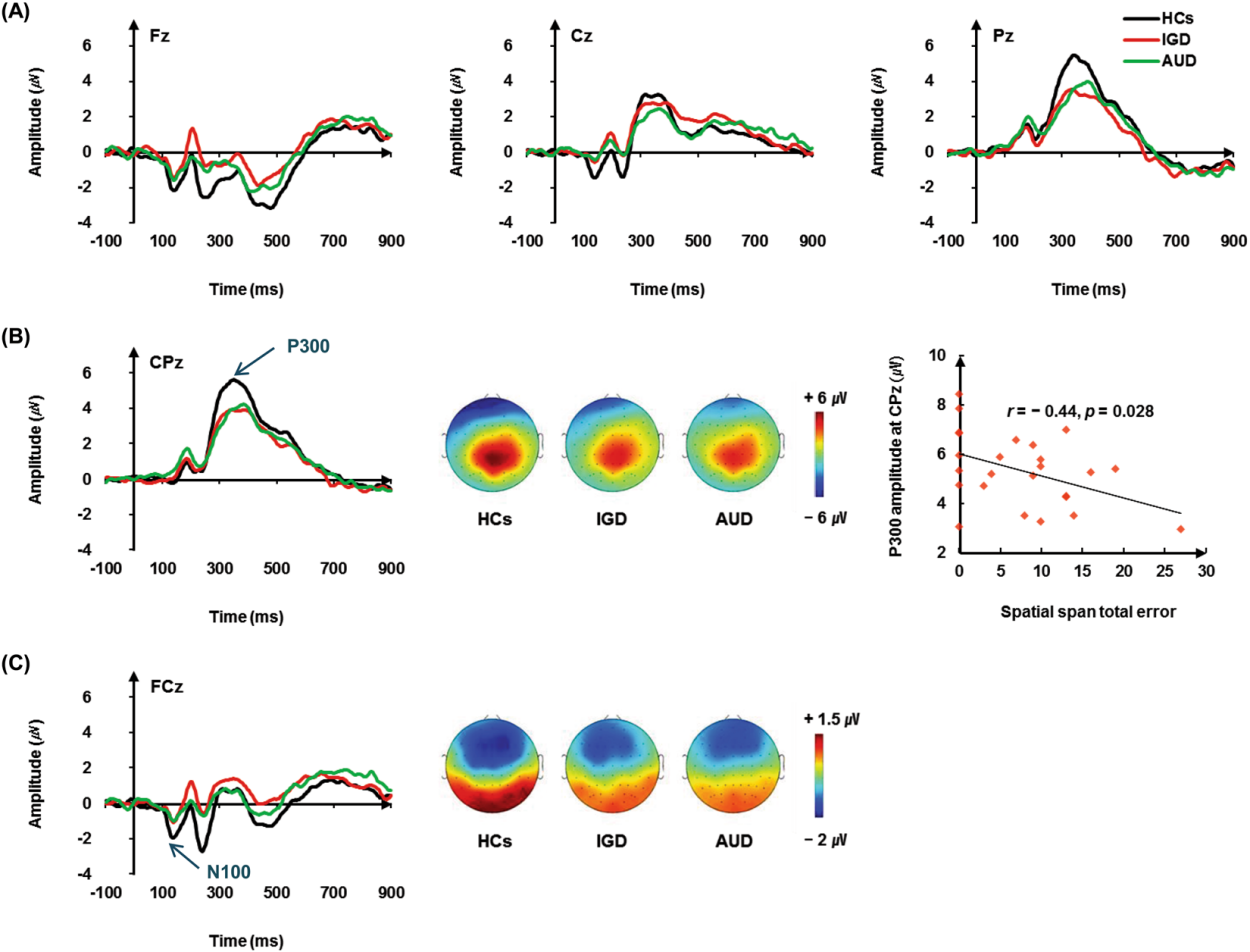
Grand-average event-related potential (ERP) waveforms and topographic maps. (**A**) Grand-average ERP waveforms over three electrode regions (Fz, Cz, and Pz) elicited by deviant tones in the auditory oddball task in patients with Internet gaming disorder (IGD) or alcohol use disorder (AUD) and healthy controls (HCs). (**B**) Grand-average ERP waveforms over CPz (left), topographic maps displaying scalp distribution of P300 amplitude in each group (middle), and correlations between P300 amplitude at the CPz and the total number of errors on the spatial span test in the IGD group (right). (**C**) Grand-average ERP waveforms over FCz (left) and topographic maps displaying scalp distribution of N100 amplitude in each group (right).

ERP values are presented in Table 2. The grand average ERP waveforms at the five midline electrode sites in response to deviant stimuli are shown in Fig. 1.

#### P300 components

A significant main effect of group (*F*_2, 70_ = 4.553, *p* = 0.014) was found for the P300 amplitude. A *post hoc* Fisher’s least significant difference (LSD) test revealed that P300 amplitudes were lower in patients with IGD (*p* = 0.022) and AUD (*p* = 0.011) than in HC subjects, whereas no difference was found between the IGD and AUD groups (*p* = 0.533). There were no significant main effects of electrode sites or interaction effects between electrode sites and group for the P300 amplitudes. In the P300 latencies, there were no significant main effects of electrode site. In the P300 latencies, there were no significant main effects or interaction effects between electrode sites and group.

#### N100 components

A significant main effect of group (*F*_2, 69_ = 3.316, *p* < 0.05) was found for the N100 amplitudes. A *post hoc* test revealed that N100 amplitudes were lower in patients with IGD (*p* = 0.012) than in HC subjects, whereas no difference was found between the IGD and AUD groups (*p* = 0.301) or between the AUD and HC groups (p = 0.333). In addition, no group effect was found in the ERP N100 values between the AUD group and the HCs. No significant interaction was observed between electrode site and group for the N100 amplitudes. Group did not show any significant main or interaction effects on the latency of the N100 wave.

### Correlations between ERP amplitudes and clinical and neurocognitive variables

No significant correlations were found between the N100 or P300 amplitudes and the clinical data in either group. The P300 amplitudes at the CPz in patients with IGD were negatively correlated with the total number of errors on the Spatial Span test (SSP) (r = −0.44, *p* = 0.028). The remaining neurocognitive data were not correlated with the P300 or N100 values in either patient group.

## Discussion

This is the first study to explore the neurophysiological features of auditory information processing and their relationships with neurocognitive functions in patients with IGD and AUD and HCs. We found that both the IGD and AUD groups showed reduced P300 amplitudes at the midline central and parietal area compared with the HCs, and the IGD also exhibited reduced N100 amplitudes at the midline frontal area compared with the HCs. The reduced P300 were correlated with a higher spatial span error rate in the IGD.

In the present study, we recorded the brain activities of all participants during an auditory oddball task to assess sensory information processing and cognitive functions. Aberrations in the ERP P300 waves in both groups of patients compared with the HCs confirmed and replicated previous robust findings of reduced P300 amplitudes recorded on the scalp surface^21, 22^. The P300 component is an endogenous, nonspecific component that is affected by effortful cognitive regulation during a given task. A decrease in P300 amplitude in response to infrequent task-relevant stimuli is consistently reported in patients with chronic AUD^29, 36, 37^. Although P300 wave amplitude can diminish in visual and auditory modalities, it is more consistently seen in the visual modality^38^. Although the majority of electrophysiological studies in patients with AUD have focused on males due to the higher prevalence of AUD in this group, it has also been demonstrated that P300 amplitude decreases at midline electrode sites in females with AUD^39, 40^. Impairment of P300 amplitude in patients with AUD does not recover after long-term abstinence^41, 42^. In addition, young boys at high risk for AUD who are not exposed to alcohol or other drugs of abuse have reduced P300 amplitudes compared with those of age-matched control subjects^43^. Other studies of individuals at high risk for developing AUD have reported smaller P300 wave amplitude^38, 44^. Thus, a P300 wave deficit in high-risk individuals may be involved in the genetic predisposition that precedes the development of AUD or related disorders, suggesting that the P300 wave is an endophenotypic marker of vulnerability to AUD.

A previous study reported that, during an auditory oddball task, patients with severe IGD manifest lower P300 wave amplitudes over the centro-parietal and parietal regions in response to deviant stimuli than do HCs, indicating that patients with IGD have difficulty processing auditory information and cognitive functioning without manifesting striking behavioral deficits^22^. In addition, although the neurocognitive functions did not significantly differ across groups in this study, we found a negative correlation between P300 amplitude at the CPz electrode site and the total number of errors on the SSP in the IGD group. In other words, an increase in the total number of errors on the SSP, which was designed to assess working memory, was found, along with reduced P300 amplitudes, in patients with IGD. These findings suggest that lower P300 amplitudes are associated with more impaired cognitive capacity in patients with IGD.

In this study, both patients with IGD and AUD manifested abnormalities in P300 wave amplitudes. These observations are congruent with the findings of previous ERP studies on patients with AUD. The decrease in P300 wave amplitude in the AUD group compared with that in the HCs was not associated with the severity of AUD. Our findings support the notion that P300 amplitude can be used as a trait marker for AUD. Similarly, no correlation was found between P300 amplitude and IGD severity in patients with IGD. These findings suggest that auditory P300 amplitude could serve as a trait marker for IGD and that patients with IGD share neurophysiological features with those with AUD, one of the SUDs.

A number of studies have focused on P300 waves in patients with AUD, but few studies have reported on the early sensory N100 component during an oddball task^45^. The N100 wave is considered an exogenous component that occurs without effortful task demands, although it can be modulated by attention. The earlier automatic N100 component depends predominantly on the physical attributes of the applied stimuli, such as modality and stimulus intensity, and is thought to mainly reflect obligatory sensory or perceptual responses after stimulus onset^29, 46^.

Contrary to our hypothesis, the auditory N100 amplitudes for target stimuli decreased at the midline frontal region only in patients with IGD. These reductions of the N100 amplitudes suggest that patients with IGD have a dysfunction in sensory and perceptual processing of auditory information, as compared to HCs. Although patients with AUD had lower auditory N100 wave amplitudes than HCs, the difference was not significant. In addition, the P300 amplitudes were not correlated with the N100 amplitudes in the IGD, AUD, or HC groups, suggesting that the N100 component is not directly associated with deficits in P300 amplitude.

Several studies have reported a decrease in visual N100 amplitudes in individuals with AUD^45^. Cohen *et al*.^29^ found that visual N100 amplitude is low in patients with AUD compared with HCs, but no difference in the auditory N100 component was observed between the AUD group and HCs. It is possible that auditory N100 is less sensitive than the visual N100 component in patients with AUD. The different N100 component results in patients with IGD and AUD may be associated with features of the auditory modality. In this study, patients with AUD developed a decrease in the auditory P300 amplitude but not in the N100 component. The findings of this study suggest that patients with AUD have deficits in P300 amplitude attributable to cognitive capacity, but they do not have impaired auditory neural processing mechanisms, regardless of the dysfunctional cognitive processing. On the other hand, patients with IGD manifested a decrease of amplitude in both the N100 and P300 components, indicating that patients with IGD might be vulnerable to dysfunction emerging from early stage of auditory information processing.

This study had several limitations. First, the study included only male participants and had a relatively small sample size, which limits the generalizability of the results. Second, the three groups were not exactly age- or IQ-matched. The AUD group was older than the IGD group, and the IQ of the AUD group was lower than that of the HCs. However, we statistically controlled for the effects of age and IQ, and the N100 and P300 amplitude-yielded group effects were not correlated with age or IQ. Further, our findings do not allow us to infer whether the decreases in N100 and P300 waves observed in patients with IGD represent a predisposition to develop IGD. Therefore, we need to investigate whether the abnormalities in the N100 and P300 components associated with the IGD group are inherited, whether they could be used to predict a high risk of IGD, whether they are a permanent effect of IGD, or whether they could be reversible after pharmacological treatment.

In conclusion, we confirmed that patients with IGD and AUD have an abnormal P300 wave component in response to a rare target tone, which supports prior studies, but we also found a negative correlation between P300 amplitude and total number of errors on the SSP in patients with IGD. This result supports our hypothesis that IGD, known as a behavioral addiction, may share P300 wave component features with AUD. Therefore, the P300 wave component may be a neurophysiological marker of IGD as one of psychiatric conditions, shared by individuals with IGD and AUD. Moreover, patients with IGD but not those with AUD showed decreased auditory N100 wave amplitude compared with HCs during the oddball ERP task. Although this change in the auditory N100 amplitudes between the IGD and AUD group is inconsistent with our hypothesis, abnormalities in the N100 wave could be a candidate of specific neurophysiological trait marker for IGD. This study will contribute to our understanding of the brain-based pathophysiological features of IGD.

## Materials and Methods

### Subjects

Twenty-six patients diagnosed with IGD, 22 patients diagnosed with AUD, and 29 HCs participated in this study. All patients were seeking treatment at the outpatient clinic of SMG-SNU Boramae Medical Center in Seoul, South Korea due to excessive participation in Internet gaming or alcohol consumption. The study was conducted in accordance with the Declaration of Helsinki. The Institutional Review Board of the SMG-SNU Boramae Medical Center approved this study protocol, and all subjects provided written informed consent prior to participation.

A clinical interview was administered by an experienced psychiatrist to diagnose IGD according to DSM-5 criteria, and Young’s Internet Addiction Test (IAT)^47^ was used to assess the severity of the disorder. We included subjects with IAT scores >50 who spent >4 h daily and 30 h per week using Internet games. The mean IAT score of participants in the IGD group was 76.25 ± 8.54. In addition, the Structured Clinical Interview for DSM-IV was used to identify past and current psychiatric illnesses. Diagnoses of AUD were based on DSM-5 criteria and were made by a clinically experienced psychiatrist. AUD severity was assessed using the Korean version of the Alcohol Use Disorder Identification Test (AUDIT-K)^48^. The mean AUDIT-K score for patients with AUD was 24.65 ± 5.23. Patients with AUD used Internet games <2 h per day and had abstained from alcohol for at least 2 weeks prior to their participation in the study to rule out acute intoxication. Abstinence from alcohol was verified by self-report and reports from caregivers. We regarded these reports as reliable because the participants attended regular follow-up visits to our outpatient clinic and showed good adherence to treatment. The HCs were recruited from the local community and had no history of any psychiatric disorder. HCs played Internet games <2 h per day and drank fewer than 14 standard drinks per week and fewer than four standard drinks per occasion. They also had no lifetime history of AUD, and their mean IAT score was 28.50 ± 8.26.

Exclusion criteria were a history of significant head injury, seizure disorder, mental retardation, or psychotic disorder. Individuals with both IGD and AUD were excluded from participation in this study. All participants were medication-naive at the time of assessment. The Korean version of the Wechsler Adult Intelligence Scale-III (WAIS-III) was administered to all subjects to estimate IQ, and only subjects with WAIS-III scores >80 were included.

### Experimental procedure

A detailed description of the experimental procedure was presented in our previous report^19^. A pseudorandom sequence of deviant stimuli (15%) and standard stimuli (85%) were presented binaurally by a STIM 2 sound generator (Compumedics, El Paso, TX, USA) for the auditory oddball task. In total, 300 stimuli were presented binaurally through earphones; the deviant stimulus was classified as a high-frequency tone (2,000 Hz) and 85-dB SPL, and the standard stimulus was classified as a low-frequency tone (1,000 Hz) and 85-dB SPL. Each stimulus had a duration of 100 msec (10-ms rise and fall times) with uniform intertrial intervals of 1,250 msec. The participants had to press a response-box button with their right hand in reaction only to a high-pitch sound. The speed and accuracy of the response were emphasized.

### EEG recording

EEG data were recorded using a 64-channel Quick-cap system (Compumedics) that referred to the linked mastoid in an isolated sound-shielded room with the ground channel located in an electrode cap between FPz and Fz. Horizontal and vertical electro-oculograms (EOGs) were recorded from electrodes placed at the outer canthus of each eye and above and underneath the left eye. The sampling rate for EEG recording was 250 or 500 Hz. The bandpass filter was set to 0.3–100 Hz. The impedance of all electrodes was <10 kΩ.

### ERP analysis

Electrophysiological signals were further processed off-line using Curry 7 software (Compumedics). Recordings were first down-sampled to 250 Hz. Data were then re-referenced to a common average reference and filtered using a bandpass frequency of 0.3–30 Hz. All EEG and EOG recordings were visually inspected to reject gross artifacts, such as those involving movement. Eye blinks and eye movements were corrected based on the artifact reduction method developed by Semlitsch *et al*.^49^. Data were segmented into 1,000-msec epochs, which included the 100 msec prior to stimulus onset. All segments with voltage > ±70 µV were automatically discarded from further processing. Trials with response times >800 msec were considered error responses and were rejected. Only those trials with correct responses at the five midline sites (Fz, FCz, Cz, CPz, and Pz) to the infrequent stimuli were averaged and analyzed. The ERP waveforms of each participant had a minimum of 30 artifact-free trials. The auditory P300 was identified as the most positive peak in a 248–500-msec time window following stimulus onset. The auditory N100 component was defined as the most negative peak in the latency range of 80–180 msec. Topographic maps were created using Matlab 7.10.0 (MathWorks, Natick, MA, USA) and EEGLAB toolbox^50^.

### Neuropsychological assessments

To evaluate correlations between the ERP values and neurocognitive functioning of each group, we administered a comprehensive neuropsychological assessment battery, including the Stroop Color and Word Test, which was used to assess attention, cognitive inhibition, and working memory^51^; the Trail Making Test, which was used to assess motor planning and cognitive shifting^52^; the three executive function subtests of the Cambridge Neuropsychological Test Automated Battery (CANTAB), a computerized neurocognitive battery; the Intra-Extra Dimensional Set Shift test (IED), which were used to assess attentional set formation maintenance and the ability to shift and flexibly allocate attention; the Spatial Span test (SSP), which was used to assess working memory; and the Stop Signal Test (SST), which is used to assess the ability to inhibit a prepotent motor response in a wide variety of clinical populations^53^. The CANTAB test is neuropsychological assessment tool to assess several cognitive abilities such as working memory, visual and spatial memory, executive functions, sustained attention and decision making in a standardized manner^54^ and was conducted using a touch-sensitive screen system that recorded error rate and latency (see http://www.cambridgecognition.com for details).

### Statistical analysis

Demographic data were analyzed with one-way analysis of variance (ANOVA) using group (IGD, AUD, and HCs) as the between-subjects factor. Patients with AUD were significantly older than patients with IGD and HCs. Patients with AUD had lower estimated IQ scores than did HCs. Analysis of covariance (ANCOVA) was used to compare the clinical, neurocognitive, and behavioral data of the groups, using age and IQ as covariates. The amplitudes and latencies of the P300 component were separately analyzed with repeated-measures ANOVAs (rmANOVAs) using electrode site (Cz, CPz, and Pz) as a within-subject factor and group as a between-subjects factor. The N100 component results from the Fz and FCz sites were subjected to rmANOVA using electrode site as a within-subject factor and group as a between-subjects factor. As age and IQ were significantly different between the groups, the rmANOVA was carried out with and without age and IQ as covariates; however, results did not differ, therefore only results with age and IQ as covariates were reported. Lower-bound corrections were applied to correct against violations of sphericity and reported the uncorrected degrees of freedom and corrected *P*-values. *Post hoc* tests with LSD were conducted to identify the direction of the group differences. ERP values were subjected to analyses of clinical and neurocognitive variables using a two-tailed Pearson’s correlation coefficient for each group separately. *P*-values < 0.05 were considered significant. All statistical analyses were performed using SPSS for Windows ver. 20.0 (SPSS Inc., Chicago, IL, USA) software.

## References

[1] Griffiths M (1997). Psychology Of Computer Use: Xlhi. Some Comments On’Addictive Use Of The Internet’ By Young. Psychol. Rep..

[2] American Psychiatric Association. Diagnostic and statistical manual of mental disorders (5^*th*^*ed*.). American Psychiatric Publishing: Washington DC (2013).

[3] Holden C (2001). ‘Behavioral’ addictions: do they exist?. Science.

[4] Grant JE, Potenza MN, Weinstein A, Gorelick DA (2010). Introduction to behavioral addictions. Am. J. Drug. Alcohol. Abuse..

[5] Choi S-W (2014). Similarities and differences among Internet gaming disorder, gambling disorder and alcohol use disorder: A focus on impulsivity and compulsivity. J. Behav. Addict..

[6] Dong, G., Li, H., Wang, L. & Potenza, M. Cognitive control and reward/loss processing in Internet gaming disorder: Results from a comparison with recreational Internet game-users. *Eur. Psychiatry* (2017). 10.1016/j.eurpsy.2017.03.004 28545006

[7] Dowling NA, Quirk KL (2009). Screening for Internet dependence: Do the proposed diagnostic criteria differentiate normal from dependent Internet use?. Cyberpsychol. Behav..

[8] Hwang JY (2014). Shared psychological characteristics that are linked to aggression between patients with Internet addiction and those with alcohol dependence. Ann. Gen. Psychiatry..

[9] Potenza MN (2006). Should addictive disorders include non‐substance‐related conditions?. Addiction.

[10] Kim H (2015). Resting-state regional homogeneity as a biological marker for patients with Internet gaming disorder: a comparison with patients with alcohol use disorder and healthy controls. Prog. Neuropsychopharmacol. Biol. Psychiatry..

[11] Ko C-H (2015). Altered gray matter density and disrupted functional connectivity of the amygdala in adults with Internet gaming disorder. Prog. Neuropsychopharmacol. Biol. Psychiatry..

[12] Dong G, Huang J, Du X (2012). Alterations in regional homogeneity of resting-state brain activity in internet gaming addicts. Behav. Brain. Funct..

[13] Lin X, Dong G, Wang Q, Du X (2015). Abnormal gray matter and white matter volume in ‘Internet gaming addicts’. Addict. Behav..

[14] Wang, H. *et al*. The alteration of gray matter volume and cognitive control in adolescents with internet gaming disorder. *Front. Behav. Neurosci*. **9**, doi:10.3389/fnbeh.2015.00064 (2015). 10.3389/fnbeh.2015.00064 PMC4367166 25852507

[15] Hong S-B (2013). Reduced orbitofrontal cortical thickness in male adolescents with internet addiction. Behav. Brain. Funct..

[16] Choi J-S (2013). Resting-state beta and gamma activity in Internet addiction. Int. J. Psychophysiol..

[17] Son K (2015). Neurophysiological features of Internet gaming disorder and alcohol use disorder: a resting-state EEG study. Transl. Psychiatry..

[18] Picton T (2000). Guidelines for using human event-related potentials to study cognition: recording standards and publication criteria. Psychophysiology.

[19] Polich J (2007). Updating P300: an integrative theory of P3a and P3b. Clin. Neurophysiol..

[20] Donchin, E., Ritter, W. & McCallum, W. C. *Cognitive psychophysiology*: *The endogenous components of the ERP. Event-related brain potentials in man* 349–411 (Academic Press, 1978).

[21] Porjesz B (2005). The utility of neurophysiological markers in the study of alcoholism. Clin. Neurophysiol..

[22] Park M (2016). Dysfunctional information processing during an auditory event-related potential task in individuals with Internet gaming disorder. Transl. Psychiatry..

[23] Porjesz, B. & Begleiter, H. *Effects of alcohol on electrophysiological activity of the brain*. (Oxford University Press, 1996).

[24] Hillyard SA, Hink RF, Schwent VL, Picton TW (1973). Electrical signs of selective attention in the human brain. Science.

[25] Knight RT, Scabini D, Woods DL, Clayworth C (1988). The effects of lesions of superior temporal gyrus and inferior parietal lobe on temporal and vertex components of the human AEP. Electroencephalogr. Clin. Neurophysiol..

[26] Coull JT (1998). Neural correlates of attention and arousal: insights from electrophysiology, functional neuroimaging and psychopharmacology. Prog. Neurobiol..

[27] Porjesz, B. & Begleiter, H. *Evoked brain potentials and behavior* 277–302 (Springer, 1979).

[28] Parsons OA, Sinha R, Williams HL (1990). Relationships between Neuropsychological Test Performance and Event‐Related Potentials in Alcoholic and Nonalcoholic Samples. Alcohol. Clin. Exp. Res..

[29] Cohen HL, Ji J, Chorlian DB, Begleiter H, Porjesz B (2002). Alcohol‐Related ERP Changes Recorded From Different Modalities: A Topographic Analysis. Alcohol. Clin. Exp. Res..

[30] Pfefferbaum A, Horvath TB, Roth WT, Kopell BS (1979). Event-related potential changes in chronic alcoholics. Electroencephalogr. Clin. Neurophysiol..

[31] Pfefferbaum A, Ford JM, White PM, Mathalon D (1991). Event‐Related Potentials in Alcoholic Men: P3 Amplitude Reflects Family History But Not Alcohol Consumption. Alcohol. Clin. Exp. Res..

[32] Polich J, Kok A (1995). Cognitive and biological determinants of P300: an integrative review. Biol. Psychol..

[33] Walhovd KB, Fjell AM (2003). The relationship between P3 and neuropsychological function in an adult life span sample. Biol. Psychol..

[34] Fabiani M, Friedman D, Cheng JC (1998). Individual differences in P3 scalp distribution in older adults, and their relationship to frontal lobe function. Psychophysiology.

[35] Kok A (2001). On the utility of P3 amplitude as a measure of processing capacity. Psychophysiology.

[36] Fein G, Chang M (2006). Visual P300s in long‐term abstinent chronic alcoholics. Alcohol. Clin. Exp. Res..

[37] Kamarajan C (2005). Alcoholism is a disinhibitory disorder: neurophysiological evidence from a Go/No-Go task. Biol. Psychol..

[38] Polich J, Pollock VE, Bloom FE (1994). Meta-analysis of P300 amplitude from males at risk for alcoholism. Psychol. Bull..

[39] Prabhu VR (2001). Visual p3 in female alcoholics. Alcohol. Clin. Exp. Res..

[40] Suresh S (2003). Auditory P3 in female alcoholics. Alcohol. Clin. Exp. Res..

[41] Porjesz, B. & Begleiter, H. *Alcohol and the Brain* 139–182 (Springer, 1985).

[42] Glenn S, Parsons OA, Sinha R (1994). Assessment of recovery of electrophysiological and neurophysiological functions in chronic alcoholics. Biol. Psychiatry..

[43] Begleiter H, Porjesz B, Bihari B, Kissin B (1984). Event-related brain potentials in boys at risk for alcoholism. Science.

[44] Porjesz B, Begleiter H (1990). Event-related potentials in individuals at risk for alcoholism. Alcohol.

[45] Patterson BW, Williams HL, McLean GA, Smith LT, Schaeffer KW (1987). Alcoholism and family history of alcoholism: Effects on visual and auditory event-related potentials. Alcohol.

[46] Näätänen R, Picton T (1987). The N1 wave of the human electric and magnetic response to sound: a review and an analysis of the component structure. Psychophysiology.

[47] Young KS (1996). Psychology of computer use: XL. Addictive use of the Internet: a case that breaks the stereotype. Psychol. Rep..

[48] Lee B, Lee C, Lee P, Choi M, Namkoong K (2000). Development of Korean version of alcohol use disorders identification test (AUDIT-K): Its reliability and validity. J. Korean. Acad. Addict. Psychiatry..

[49] Semlitsch HV, Anderer P, Schuster P, Presslich O (1986). A solution for reliable and valid reduction of ocular artifacts, applied to the P300 ERP. Psychophysiology.

[50] Delorme A, Makeig S (2004). EEGLAB: an open source toolbox for analysis of single-trial EEG dynamics including independent component analysis. J. Neurosci. Methods..

[51] Stroop JR (1935). Studies of interference in serial verbal reactions. J. Exp. Psychol..

[52] Reitan, R. M. *Trail Making Test: Manual for administration and scoring*. (Reitan Neuropsychology Laboratory, 1992).

[53] Robbins T (1994). Cambridge Neuropsychological Test Automated Battery (CANTAB): a factor analytic study of a large sample of normal elderly volunteers. Dement. Geriatr. Cogn. Disord..

[54] Owen AM, Downes JJ, Sahakian BJ, Polkey CE, Robbins TW (1990). Planning and spatial working memory following frontal lobe lesions in man. Neuropsychologia.

